# Caring for Women of Color: Community-Based Doulas’ Strategies in Hospital Birth in Los Angeles

**DOI:** 10.1080/01459740.2025.2495633

**Published:** 2025-05-04

**Authors:** Kim Sigmund

**Affiliations:** Department of Anthropology, University of Amsterdam, Amsterdam, The Netherlands

**Keywords:** Community-based doula, intersectional precarity, Los Angeles, reproductive justice, transformative agency, white medical gaze

## Abstract

In the United States, women of color experience worse pregnancy and birth outcomes than white women. Likewise, many women of color report facing discrimination from perinatal health providers, and many experience precarity that can negatively impact birth experiences and outcomes. In this context, more women of color now embrace the use of community-based doulas. Using ethnographic data, I argue that community-based doulas, as members of the communities in which they offer their services, are uniquely able to negotiate the tensions between their clients and biomedical birth practitioners to engender acts of transformative agency and forward the cause of reproductive justice.

On a cold February morning in 2020, I was talking with Guadalupe, a program coordinator and case manager at the Maternal Aid Center (MAC) in downtown Los Angeles, as we left the weekly breastfeeding and nutrition class for Central American migrant mothers. “I would really like someone to come and talk to the women about their options, like how they can have a midwife or even a doula with them when they give birth.” Guadalupe shared with me as we stood in the hall of the dingy old building that housed MAC’s services and support staff. MAC was one of the main non-governmental organizations in Los Angeles that specifically offered maternal health education and support services for low-income women in the community, most of whom were Central American migrants.

As Guadalupe and I talked over the buzz of the fluorescent lights, I asked if she thought many of the Central American women at MAC would prefer to have a doula with them when giving birth, and Guadalupe quickly affirmed yes. A Guatemalan migrant herself who usually spoke in a kind yet authoritative voice, her voice filled with frustration and indignation as she explained to me that many of the women she worked with as a case manager at MAC wanted to work with a non-biomedical birth attendant, but they were not covered by Medi-Cal^1^ which paid for most of MAC’s undocumented clients’ perinatal costs. Additionally, Guadalupe said that she had noticed that the Central American migrant women she worked with were afraid to ask for an alternative care provider because they feared being judged by their doctors because of their social and economic positions. I asked her what she meant, and she explained “they won’t tell their doctors if they use other forms of care because the doctors shun it.” Guadalupe said “in my opinion, the western doctors in the United States are not open to other ideas. They are just flat, just closed and don’t want to consider other options, so the women don’t even mention anything else [when they speak to their OB/Gyn].” This was frustrating to Guadalupe because the migrant women “don’t know their rights when it comes to pregnancy and birth … they don’t know that they even have options in terms of what kind of birth to have, the doctors present their preferred option as the only option … and many times the doctors don’t speak Spanish.” Likewise, she knew that many of the hospitals did not allow doulas inside because the doctors did not like having someone “push back” against the advice that they give to the pregnant women or birthing mothers. Guadalupe continued:

I have gone with women to the doctors and been in the delivery room with them, and I have seen how the doctors change and offer the women better care when they learn who I am and see that I can speak both English and Spanish. If I’m not there, they get really bad care.

This conversation I had with Guadalupe demonstrates multiple elements that impact the birth experiences of racialized, marginalized women in the United States (US). First, it hints at the precarious positionality of Central American migrant women in the United States that can influence their needs and desires during birth. When I collected this data in 2020, Central American migrant women were eligible under Medi-Cal for full prenatal and birth coverage, plus 60 days of postpartum care (DPSS 2024). However, their lack of legal documentation, language barriers, economic insecurity, lack of knowledge about their rights to care, and implicit racial bias by medical staff negatively impacted both their willingness and capacity to access the care they were eligible for (Morey 2018; North 2019; Philbin et al. 2018; Vedam et al. 2019). Secondly, the conversation demonstrates how biomedical practitioners may be unable to fully recognize the precarities or needs of their racialized, marginalized patients because they are “closed off” to the lived realities or alternative wishes of their patients. Lastly, Guadalupe’s story suggests that in Los Angeles, social service providers recognize that the support of a doula is useful for mitigating the unequal and inhospitable relationship between patients and biomedical providers and advancing the cause of reproductive justice in birth in the United States (Ellmann 2020; HMHBA 2023). In this article, I demonstrate how community-based doulas (CBDs), who share communal relationality with their clients, engender healthy, safe, and respectful birth experiences for racialized, marginalized women in Los Angeles. I argue that CBDs accomplish this through patient advocacy, reframing the birth experience for their clients, and through acts of transformative agency.

## Reproductive justice and racialization

Reproductive justice is defined by Loretta Ross and Rickie Solinger as “the right not to have a child; the right to have a child; and the right to parent children in safe and healthy environments” (Ross and Solinger 2017:9). In their estimation, this requires “access to specific, community-based resources including high-quality health care, housing and education, a living wage, a healthy environment, and a safety net for times when these resources fail” (Ross and Solinger 2017:9). However, many Black and Latina women in Los Angeles do not have access to one or more of these resources due to processes of racialization and marginalization.

Black women have been racialized by conservative politicians in the United States as criminals, mothers of future criminals, women with “unruly bodies” who have more pregnancies than white women, and “welfare queens” who are believed to have children in order to take advantage of the US welfare system (Bridges 2011; Ross and Solinger 2017; Sufrin 2017). Likewise, biomedical practitioners stereotype Black women as hardier than white women, therefore needing less pain relief in birth; and as troublesome patients who overreact and do not require or deserve the same level of health care attention as white women (Bridges 2011; Davis 2019a), leading to discrimination in health care (Davis 2019b; Salinas et al. 2022).

Similarly, conservative politicians and anti-migrant groups have racialized Latina women as “illegal” – regardless of migration status – due to their skin color, and a “threat to the nation” based on stereotypes of their sexuality, reproduction rates, and fertility (Chavez 2013:73; De Genova and Ramos-Zayas 2003; Morey 2018). This leads to discrimination when they seek out health care, and they face racial profiling by the police and Immigration and Customs Enforcement (ICE) (De Genova 2002; Ross and Solinger 2017; Viruell-Fuentes et al. 2012). The racialization of Black and Latina women leads to marginalization when the state enacts racist policies that limit their access to resources such as legal, medical, economic, and social support (Abrego and Menjívar 2011; Menjívar and Abrego 2012; Ross and Solinger 2017). In California, Black and Latina women – especially Latina migrants – are often unable to access adequate perinatal health care (March of Dimes 2024), live in neighborhoods without safe or affordable housing, live in poverty, and lack adequate or reliable community support (Singhal et al. 2017).

## Precarity

Together, racialization and marginalization create various and intersecting barriers to care and states of vulnerability which coalesce to create a precarious environment for Black and Latina women (Andaya 2019; Sigmund 2023). Ramirez et al. (2021:5) define precarity as an “existence defined by vulnerability, unpredictability, and insecurity, be it in the realm of work, housing, health, or other aspects of life … ”. They later state that “ … for people who are socially, economically, or politically vulnerable, [a lack of stable income or access to health care] exacerbates an already precarious situation, as their risks are worsened by both a lack of [legal] protection and a lack of control in their nonwork lives” (2021:5). More importantly, precarity is a state of persistent insecurity, a form of regulation that is induced when the state deems certain marginalized and racialized traits a threat to society, marking the lives of those who embody those traits less valuable than the socio-economic majority (Butler 2009). For women who embody multiple identifiers that mark them as undeserving of care by the state, such as being a person of color, lacking citizenship status, low income, and lacking English language skills, state-induced socio-economic precarity intersects with implicit biases held by those in power to shape and reinforce the social inequalities in their lives (Crenshaw 1991; Hill Collins 2015).

Alongside state-induced precarity, recent research has demonstrated that it is difficult for marginalized women to experience a safe, healthy, and respectful birth in US birth spaces due to public policies that have limited the quality and types of perinatal care marginalized women can access and the one-size-fits-all standardization of obstetric care that disregards the socio-economic, cultural, and psychological needs and desires of migrant women and women of color (Hamm et al. 2022; Masters et al. 2023; Morgan 2019; Ross and Solinger 2017). Specifically, I argue that non-racialized biomedical practitioners view their patients through a white medical gaze, which may increase their incapacity to attend to these needs. Expanding Foucault’s concept of the “medical gaze” (Foucault 2003; Misselbrook 2013), I suggest that a white medical gaze may lead non-racialized biomedical practitioners to filter out important information about their patient’s racial identities that they deem irrelevant so that they can fit their diagnosis more neatly within the biomedical model. Such disregard or incapacity is visible specifically in the evidence of ongoing racial biases in Californian hospitals and birth spaces that disregard women of color’s wishes or concerns during pregnancy and birth (Arteaga et al. 2023). Indeed, as the above conversation with Guadalupe alluded to, it is difficult for racialized, marginalized women in Los Angeles to truly receive fair and unbiased birth support within the biomedical system.

## Birth in the United States

Although biomedical birth leads to safe and healthy birth outcomes for many women and babies, qualitative research has demonstrated that because of institutional racism and socio-economic barriers to equitable perinatal care, many women of color do not have good birth experiences in US hospitals (Bridges 2011; Davis 2019a; Ellmann 2020; Vedam et al. 2019; Wang et al. 2021). In the United States, Black and Latina women experience higher maternal mortality rates than white women: from 2019 to 2021 Black women in California died at a rate of 49.7 per 100,000 live births versus 17.7 for Latina and 14.0 for white women (CDPH 2024; Wang et al. 2021). Additionally, many Black and Latina women have come forward about mistreatment and trauma they received within the biomedical birth system (Davis 2019a; Vedam et al. 2019). In Los Angeles, the Office of Women’s Health noted that 54 percent of Black women and 38 percent of Latina women had experienced discrimination during birth, of which 40 percent of Black women and 15 percent of Latinas felt that this discrimination was due to the color of their skin (Singhal et al. 2017). Additionally, 79 percent of Black women and 66 percent of Latinas experienced stressful situations during pregnancy and around 20 percent did not have social support during their pregnancy (Singhal et al. 2017).

## Doula birth care

Conversely, recent studies have shown that doula-supported births help women of color feel supported and that they have received respectful care during birth (Arteaga et al. 2023; Mallick et al. 2022). Doula birth support is also associated with a lowered rate of pre-term birth, shorter labor, decreased need for medications, fewer cesarean sections, higher rates of breastfeeding initiation, less anxiety, and less postpartum depression (Ellmann 2020; Kozhimannil et al. 2013; Steel et al. 2015). Additionally, recent research has discussed how doulas offer much needed continuous care during birth (Steel et al. 2015), “empower” women to have some control over the birth process (Henley 2020) and ensure women can give informed consent during birth (Ford 2021). However, most existing research on the importance of doula care in the United States has failed to discuss the distinction between what one of my interlocutors called “conventional doulas” and community-based doulas (CBDs), a distinction which I demonstrate is important for understanding the benefit that having a community-based doula can have on birth experiences and outcomes for women of color.

Historically, most conventional doulas in the United States were white women who mostly worked with middle and upper-class white women (Arteaga et al. 2023; Hunter 2012). However, recent years have seen a shift in interest for women of color to seek out doula care for their pregnancy, birth, and postpartum experiences. In the 2018 Listening to Mothers in California survey (Sakala et al. 2018), 66 percent of Black and 56 percent of Latina women in California said they were interested in using a doula for their next birth. This interest has likely increased the desire for community-based doulas in Los Angeles. Community-based doulas (CBDs) usually identify as Black, Indigenous, and people of color (BIPOC). Unlike conventional doulas who may receive more general birth support education, CBDs are trained to provide prenatal, birth, and postpartum support to pregnant women within their own ethnic group or other marginalized communities (Herriott et al. 2023; HMHBA 2023; Peprah-Wilson and Riley 2023). Their aim is to create safe birthing spaces by recognizing racist treatment and advocating for their clients’ needs, normalizing the body’s natural birthing process, re-inscribing community practices and knowledge into birth by supporting women’s use of items such as rebozos, and ensuring that “disadvantaged” and “vulnerable” populations can access birth support (HMHBA 2023; Peprah-Wilson and Riley 2023; Salinas et al. 2022).

Existing literature acknowledges the importance of CBDs for culturally responsive care, i.e. care that respects a person’s cultural traditions, values, and needs (Darvish et al. 2024; Khaw et al. 2023), especially for Black women, migrants and refugees (Akhavan and Edge 2012; Arteaga et al. 2023; Bohren et al. 2020; Khaw et al. 2022). Recent qualitative research published on CBDs in the United States explores the themes of agency, patient choice, and empowerment (Arteaga et al. 2023; Darvish et al. 2024; Kett et al. 2022; Rivera 2021). However, anthropological research linking community-based doula birth support to transformative agency in the birth space is an underexplored area. Transformative agency comes about when patients and their advocates utilize ruptures and conflicts within health-based settings in order to negotiate changes and “collective transformations” to the health system in order to improve patient care (Béhague et al. 2008; Niy and Diniz 2023). While Béhague et al. (2008) demonstrated how transformative agencies can be engendered for marginalized birthing women through the support of an advocate (i.e. a family member or friend), this concept has yet to be applied to an analysis of doula work, nor how transformative agencies may promote reproductive justice. Therefore, in this article, I add nuance to the growing body of literature in the social sciences that demonstrates the essential roles of doulas in birth care (Adams 2022; Chautems 2022; Gutschow and Davis-Floyd 2021; Searcy and Castañeda 2021). I also expand upon existing research on reproductive (in)justice (Bridges 2011; Davis 2019a; Ross and Solinger 2017) by demonstrating how community-based doulas (CBDs) fill a specific need in the birth care of Black and Latina women by creating space for transformative agency when the needs and desires of women of color are overlooked by biomedical practitioners whose vision is limited by the white medical gaze.

## Methods

The data in this article is based on long-term fieldwork in Los Angeles, USA, from October 2019 to September 2020, with follow-up data collection completed November -December 2021. Pre-COVID, data collection included participant observation at the maternal aid center and at a public hospital in Los Angeles, which aided me in broadly contextualizing the themes discussed in this article. Once the pandemic began, my methodology shifted toward digital interviewing and web-based data collection. It was during the pandemic that I contacted community doula organizations in Los Angeles and put requests for interviews with doulas on social media birth worker pages. Through this, I connected with multiple doulas and doula organizational heads. I connected with biomedical practitioners (obstetrician-gynecologist and labor and delivery nurses) through my role as a research assistant in a local perinatal care research collective in Los Angeles. I completed semi-structured ethnographic interviews with five community doulas (three Black, one Latina, one white), the heads of two local doula organizations who are also trained doulas (one Black one white), the head of a hospital-based doula program (Latina), one Ob/Gyn (Asian) and three labor and delivery nurses (two white, one Latina). All interlocutors have been pseudonymized and certain defining characteristics omitted to protect their privacy.

I held all interviews virtually over video conference or the phone, and all participants gave their verbal consent to take part. These ethnographic interviews allowed me to explore multiple themes surrounding birth work, experiences with clients/patients of different demographics (no identifying information was given in respect of patient privacy) and the unique role that community-based doulas play in birth care in Los Angeles. Digital meeting times allowed my informants to schedule their interviews at times when they could ensure their own comfort and privacy, including when they were driving or evenings and weekends when they could speak freely within their own homes. Due to this, respondents appeared to feel comfortable to answer my questions and openly discuss their experiences with birth care. However, virtual data collection did limit the amount of wider contextual data and hands-on understanding about what it is like to attend a birth that in-person ethnographic data collection would have engendered. To account for this limitation, I held follow-up interviews with three interlocutors to explore themes specifically related to community-based doula work and reproductive justice in Los Angeles. I gathered information on the role and importance of CBDs for women of color from CBD organization websites, and by visiting local NGOs and clinics that offered services to marginalized women.

## Biomedicine, biases and the white medical gaze

In late 2020, I was talking to Nancy, a white Labor and Delivery Nurse (LDN), over Zoom after her 12-hour shift had ended at a Los Angeles hospital. Still wearing her scrubs, she spoke enthusiastically about the labor and delivery program at her hospital; and when I asked her to tell me what the labor and delivery process was normally like in her hospital she replied with pride:

We have … an obstetrician in-house 24/7, we have an anesthesiologist and neonatologist on-staff for labor and delivery 24/7. We feel very protected, there is always someone to call for help. We have decreased our bad outcomes by having help readily available at all times. We also have a blood transfusion program and postpartum hemorrhage carts in Labour and Delivery. Because hemorrhage is one of the leading causes of maternal morbidity and mortality, we have a blood fridge on the floor for any dire emergency … All of these changes and programs have decreased our bad outcomes.

As we spoke, I noticed that Nancy’s responses focused on discussing the physical outcomes for a healthy birth, she did not focus on the mothers’ feelings or experiences. When I later asked about how she managed different ethnic or cultural wishes that patients might have for their births to see whether she took any other variables into account when discussing good birth outcomes, she proudly told me that her staff does extensive cultural bias training.

Nancy’s response aligns with recent policy changes in the United States that try and ensure hospitals offer more “patient-centered care” (Becker et al. 2014) and in California to tackle institutional medical racism through mandatory cultural competency and implicit bias training (Mitchell 2019). Cultural competency training teaches biomedical practitioners to listen more carefully to the cross-cultural aspects of their patient’s medical stories. However, it does very little to teach biomedical staff to address the intersectional relationship between health and the social, economic, and political systems that impact health for marginalized groups (Metzl and Hansen 2014). Additionally, the strict time limits placed on patient–provider interactions (Andaya 2019) alongside the white medical gaze that non-racialized biomedical providers may utilize in order to filter out information that they deem erroneous to the provision of health care, leave little time or inclination for doctors or nurses to ask about potential racial, socio-cultural, linguistic, and economic precarities that might impact their patients’ birth outcomes (Metzl and Hansen 2014).

After speaking with Nancy, I interviewed another white LDN called Amy from the same hospital. As we discussed her role in birth care, I asked her if she saw any difference in the birth experiences for women in different economic or ethnic groups. She paused, looked past me for a moment while she thought, then responded:

I don’t know, across ethnic groups people seem to respect the medical profession. Hispanics it’s to a fault sometimes, they respect the medical profession sometimes too much I feel, they don’t question things. I don’t know if it’s a lack of education? Or that they are coming from a culture of respect? I don’t really know. The higher-class white people tend to have more of a voice, they are more empowered to use it and advocate for themselves … Maybe I’m naïve, but it seems to me that … everyone gets the same prenatal care … I think LA has done a good job to make sure that everyone can access care. I know that there are some who are critical of our health system and claim there are biases and that we don’t give equal service to all socio-economic groups, but I don’t see any lack of care …

Wanting to know more about what she meant by this, I asked her how she specifically handled issues of cultural or ethnic difference with her patients, and she responded “It’s a case-by-case basis when dealing with patients from different backgrounds. Like some [Muslim] women don’t want any men in their room due to cultural reasons, but that is hard, we can’t guarantee that because all of our anesthesiologists are men … We can’t change our staff for just a few women, but we do what we can to make everyone comfortable.”

These quotes demonstrate how the white medical gaze that non-racialized biomedical practitioners direct onto their patients can lead to a lack of visibility and comprehension for the complex socio-economic, racial and gendered precarities that birthing women of color carry with them when they enter a hospital to give birth. A white provider may not recognize that they are giving a patient of color inequitable or racially biased treatment if they believe that the health care system offers equal access to care. A Muslim woman who does not want a man present during her birth may well face serious cultural and familial backlash for calling her modesty into question (Rabin 2010). A Latina (Hispanic) woman might not feel comfortable questioning her doctor’s advice who she does not have a trusting relationship with, especially if she does not fully understand what their advice is due to a language barrier (Julliard et al. 2008). Here, it appears that cultural competency training is not enough to overcome the racial biases and myopic views of birth that a white medical gaze perpetuates, making it more likely that non-racialized biomedical providers will lack the capacity to see and mitigate the multiple and interlocking precarities that influence maternal health and patient needs in the delivery room. As I will further elucidate below, it is these types of negative experiences that may be prevented with the care of a community-based doula (CBD) (Ellmann 2020; Gutschow and Davis-Floyd 2021), which may improve the likelihood that a woman of color will give birth in a healthy, safe and respectful environment.

## Community doulas for reproductive justice

Community-based doula (CBDs) may be particularly adept at caring for birthing women of color because they themselves are part of the same racial and/or marginalized communities as their clients (Rivera 2021). During fieldwork, I interviewed Kelly, a Black woman and the head of a Los Angeles-based CBD organization, twice. Sitting in her sunny kitchen one morning as we talked over Zoom, I asked her if she could explain how she thought that CBDs differed from conventional doulas. She explained:

community doulas typically have … high levels of cultural sensitivity and congruence. [So] they’re going to get better outcomes with the communities that they’re serving because they’re serving communities that look like them and they have shared lived experiences and we have shared cultural experiences. And there’s a safe space for the birthing families to not experience the harm that they might be experiencing from the Western and medical industrial complex, so it’s like home, you know, it’s feeling more like home. And it’s very important that the caregivers are looking like those you’re giving care to.

Kelly’s use of the term “cultural congruence” (Schim et al. 2007) made clear that in her experience, CBDs are better able to understand the cultural preferences, worldview, choices, and decision-making processes of their clients because they are members of the same racial communities and cultures as their clients. And as I will further elucidate below, it is this relatedness that makes CBDs especially capable of advocating for their clients’ needs. In the following sections, I will discuss how CBDs relate to their clients on a deeper level than conventional doulas specifically because they come from the same communities, and what this means for the advancement of reproductive justice in birth for communities of color in the United States.

### Sharing community

In August 2020, I began to understand this deeper doula-client connection during a phone interview I had with a Black CBD named Monique while she was driving in Los Angeles’s omnipresent traffic. Monique was frustrated about doulas not being designated essential workers during the COVID-19 pandemic. Three of her recent clients (two Black, one Latina) had given birth by cesarean section, and she felt that this was because she had not been able to attend the birth and help her clients in person. Monique responded to this, telling me:

I feel that doulas are not treasured, we are seen as a nuisance … it doesn’t feel like doulas are welcomed into the birthing room of the hospitals anymore … one of my recent clients is a 16-year-old, and her doctors tried to get her to have a c-section at 36 weeks but I was able to advocate for her … she was able to lean on me and my power to tell her that she had the right to say no, and she said no. She went to three different hospitals and they all tried to cut her open. They were taking advantage of her ignorance, in my opinion. They said she was “at risk” but they never named an actual medical reason …

Monique went on to tell me that this specific client was Black and living in the foster care system, and Monique was her only support person during birth. In the end, they were able to avoid her having a c-section and Monique was happy that her client and the baby were healthy, that the mother had been able to avoid unnecessary medical interventions, and that she had been able to make her own decisions about how she wanted to give birth.

Monique’s client had multiple needs which stemmed from her unstable home environment and lack of familial support – general precarities that made her life unstable, and her lack of knowledge about her right to say no to medical authority – which made her more directly vulnerable in her interactions with biomedical practitioners. Together, these precarities were compounded in her interactions with biomedical staff because of her young age and race, which Monique saw as factors that biomedical staff tried to “take advantage of” to “rush people through” the hospital, a common complaint CBDs voiced to me during COVID. Without having Monique present to advocate for her with various biomedical practitioners who may have viewed their patient through a gaze tinted with implicit biases about her skin color and socio-economic position, she may have received unnecessary medical care that could have led to birth trauma or a poor birth outcome. In this situation, having a CBD present who shared a racial background and who was able to advocate for her against what the CBD perceived as racial and class discrimination appeared to be essential to this woman giving birth in a healthy and safe environment.

As Monique told me during her interview “Most [of my clients] are African American … I don’t tend to work with white women … I don’t feel like I relate to them. I feel that I relate more with women from the same background as me.” For Monique, as for the rest of the CBDs I spoke with, sharing a racial and cultural background with her clients helps her relate more to them and better understand their needs. Laura, a first-generation Latina migrant and CBD echoed this sentiment. She felt that because she was a migrant herself, she had knowledge of both cultural practices done abroad and cultural practices in the United States, which she felt help her relate to Latina clients and better support them if they choose to follow Latina birthing practices such as using a faja or following *la cuarantena* to rest for 40-days after childbirth.

This resonates with Kelly’s understanding of one of the unique strengths that CBD have: CBDs serve clients who share their racial identities as well as their lived and cultural experiences. In this way, not only does the CBD understand shared cultural norms because of their own personal experience with those norms but they also have likely experienced the same disparities or precarities as their clients. For example, CBDs who are also mothers may have also experienced harm from the biomedical model, so they have an embodied knowledge of how the biomedical system impacts women of color’s experiences in birth. As Tess, a Black CBD shared with me, as an African American woman she noticed more of the mistreatment that her clients experienced because she herself had experienced mistreatment and racism as a Black woman in the past. Due to her own past experiences and the time she spent developing relationships with her clients before they gave birth, she was able to see how her clients would immediately be “on guard” in the presence of their biomedical practitioner and she knew that this was because they had experienced medical mistreatment in the past. As she said,

many women of color and low-income people are already expecting unfortunate treatment when they go to the doctor, so they are already more on guard. They expect medical interventions like induction and see it as normal to have Pitocin or balloons. And for many African Americans, it’s a trend in their family or culture, but it’s not the norm in other [socio-economic statuses] or races, like for white women. We African American women notice these differences, and I see it more frequently in Black or Hispanic women.

Through building a relational link prenatally with their clients which then continues into the birth, CBDs try to ensure that their clients feel that there is someone at the birth whom they can trust while also gaining insight into the social and economic precarities their clients face ahead of the birth. With this relationality, CBDs can see when interactions between biomedical staff and patients might be leading toward tensions that could lead to poor treatment and poor birth outcomes, and with this knowledge they can attempt to mitigate potential harm through patient advocacy.

### Advocacy

Advocacy is one of the core strategies all doulas (conventional and CBD) practice in their work to aid women to have a positive birth experience and good birth outcomes (Salinas et al. 2022). However, for CBDs advocacy work is rooted in responding to racial and class discrimination, rather than predominately trying to avoid excessive medical intervention as in conventional doula work. According to Kelly, CBDs have often experienced discrimination themselves, making them more aware of and invested in advocating for their clients to mitigate potential discrimination in the birth space than a conventional doula might be. As she noted:

[B]ecause the community doula is typically from your community and of your cultural background or ethnic background, you’re going to feel more apt to trust that relationship and share what your needs are. That way, that doula can advocate for you and be present to you and the need that aligns with your family’s desires. And so that birth satisfaction now is being created. Because now you’re feeling heard … you’re feeling cared for as opposed to just another number in an appointment system.

This appears to be especially important because, as mentioned by the CBDs I spoke with, there is a disparity in the information and treatment their racialized clients receive from biomedical staff versus white women. As CBD Stephanie told me: “I have seen this disparity play out when Black women are told ‘not to bother’ getting a lactation consultant from nurses, or when one medical provider called [Child protection services] on a mom because they felt like she was struggling with breast feeding.” Such disparities lead CBDs to strongly vocalize their clients’ wishes and needs to medical staff who may not be aware that they are treating their patients of color differently than they would a white patient.

For example, Black CBD Janet attended the birth of a Black woman who needed her doula to advocate for her. The client wanted to have a natural birth and a vaginal birth after cesarean. The woman’s brother had overdosed on fentanyl, so she wanted to avoid any pain-relieving medication and to go through labor as much as possible without interventions. But according to Janet, the medical staff kept pushing interventions on the woman. Janet said “they came in there like sharks and they tried to force her to get a C-section early on in the labor. She dilated five centimeters and then her labor slowed.” Seeing this and knowing about her client’s wishes and past experiences, Janet advocated for the woman with the doctors, asking for time to try different techniques to re-start labor. Janet used music, massage, and different labor positions to help and support the couple and relax them. After 18 hours of labor and no progression, the woman finally agreed to get a C-section. Afterwards, the family told Janet they were very happy with the advocacy she did for them, and Janet happily added that the baby was healthy and everyone was pleased with the birth outcome – despite the needed medical interventions. As Janet perceived it, having a strong awareness of differential treatment received by her Black client made her acutely aware of situations when biomedical practitioners were overlooking the patient’s needs and desires. In this context, Janet was able to use this space of tension between the biomedical staff’s desires and her client’s desires to negotiate for her client’s agency by asking the biomedical staff to hold off on pushing interventions too quickly.

### Re-framing the situation

During birth, CBDs may find that their clients struggle to understand what they are experiencing, how they are being treated in the hospital, and why their birth is progressing a specific way. In this context, CBDs can re-frame the situation to help their clients better understand what is happening and why. According to Kelly, “re-framing” is about centering the mother and her experiences during birth by de-centering the biomedical focus in order to focus on the mother, her autonomy in birth and her desires and needs. As Cathy, the white head of another Los Angeles CBD organization, explained:

If we’re dealing with minority women, they have a lot of social and environmental stressors in their lives. If there are any racism stressors or anxiety, the doula can help with that, they can ameliorate the impact of the stress on the women. They help the women to relax and talk with them about what they’re feeling and help them to reframe and trust the process. For minority women there are social and environmental stressors, like poverty, racial discrimination, etc. the doula can be a buffer for these stressors, be someone for the mom to talk to and help her reframe the situation, and give her a safe space to come to.

By re-framing or centering the mother, CBDs may help their clients to shift their focus away from outside stressors that could negatively impact birth by re-focusing the woman’s energy on her own body and the birth process so that the client can trust that their birth is progressing normally. Additionally, Cathy noted that by being a “buffer” for the social and environmental stressors they face, CBDs may help the client to re-focus her energy on the positive aspects of the birth while the CBD is advocating for the woman. By being aware of the “social and environmental stressors” – i.e. the precarities – that their clients face, the CBDs may mitigate the negative impact that racial discrimination and elevated stress hormones following poor experiences with medical staff can have on the birth experience of women of color by re-focusing the energy onto the mother and her own role within the birth. In this way, CBDs may create more space for their clients to make their own decisions about how to move forward in their births, even if the birth experience shifts and comes to require different forms of support or intervention, as we saw in Janet’s story.

Through this deeper understanding of the specific precarities, potential difficulties and discrimination that their clients face when they enter the birth space that CBDs benefit from by sharing community membership with their clients, CBDs appear to be working toward changing the wider biomedical perinatal health system by centering women of color in the birth space, instead of maintaining the biomedical focus on health outcomes that leave little space for understanding the more complex realities of their patients’ lives that have an impact on birth experiences and outcomes. Through this act of transformative agency, community-based doulas advance the cause of reproductive justice by facilitating a safer birth environment in which their client’s needs are respected and attended to.

## Conclusion

I have demonstrated how community-based doulas (CBDs) may engender healthy, safe, and respectful birth experiences for racialized, marginalized women in the United States. Through ethnographic data, I have shown that CBDs may be particularly adept at assisting women of color in childbirth because they originate from and are based within the communities that they serve – giving them unique access to and understanding of their clients’ needs when giving birth. Due to their communal relationality with their clients, they appear better able to understand the concerns, fears, and past experiences of discrimination that negatively impact the birth experiences and outcomes for women of color because many times they have experienced the same discrimination, concerns, and precarities that their clients are experiencing in their own lives.

Through ethnographic vignettes, we see that CBDs help to bridge a gap between the goals that biomedical care providers have regarding birth and the lived experiences and precarities faced by their clients. While biomedical birth support has saved countless lives through medical interventions and patient-centered care, due to a white medical gaze and ongoing implicit biases in medical care, non-racialized biomedical practitioners may not be capable of fully recognizing the intersectional precarious states and barriers to health that women of color live with in the United States today. And while conventional doulas also greatly improve the birth experiences and outcomes for many women, if they do not share the same socio-ethnic or racial backgrounds as their clients, they may not have experience recognizing the precarities or discrimination experienced by the women of color they work with. This lack of relationality limits their capacity to effectively advocate for the needs of their racialized, marginalized clients.

Research has demonstrated time and again how these precarious states and barriers to accessing adequate and equitable health care negatively impact pregnancy and birth outcomes for racialized, marginalized women (Davis 2019a; Liese et al. 2021; Steel et al. 2015; Vedam et al. 2019). While California has focused on implementing cultural competency training and other practices to improve birth outcomes for women of color, Black and Latina women continue to experience worse birth outcomes (CMQCC 2024; March of Dimes 2024). Clearly, cultural competency and conventional doula care do not do enough to counteract the precarity or discrimination experienced by Black and Latina birthing women. Indeed, multiple social scientists and activists have noted that racialized, marginalized women experience obstetric racism and racist mistreatment in US hospitals (Davis 2019a; Ross and Solinger 2017; Searcy and Castañeda n.d.); and high percentages of Black and Brown women report receiving unfair treatment in US birth care based on their race or ethnicity (Nguyen et al. 2023; Sakala et al. 2018:12, 64).

Racialized, marginalized women who are giving birth in US hospitals deserve the support of people from their own communities who can serve as a mediator between themselves and biomedical practitioners during birth. They need to know that someone is caring for both their birth experiences and their lives outside of the hospital. When a woman of color is supported by a CBD who understands her background, her lived experience, her everyday concerns, and how the medical-industrial complex can cause harm to women who look like her, the CBD may be able to facilitate a healthy and safe environment for that woman to birth within.

As I have demonstrated, it is these characteristics that make CBDs uniquely capable of recognizing and mitigating the intersectional precarities their clients face, allowing them to advocate for their clients’ needs and re-center the mother within the birth process. Through their role, it appears that CBDs create space for transformative agency within the birth space by negotiating tensions between the needs of women of color and the biomedical birth team through their advocacy work and by re-centering the mother in the birth process. These negotiations, which may seem small on an individual basis, can collectively lead to wider systemic change. By advocating for the rights and needs of women of color, CBDs may influence individual hospital policies to be more attuned to the needs of women of color or shape future implicit bias training. Through this, CBDs can advance the cause of reproductive justice by shifting the US birth space into a more just, respectful, and safe environment for all women of color.

I have also expanded the literature on transformative agency by linking it to reproductive justice through the birth work of community-based doulas. Likewise, through an in-depth discussion of the unique role that community-based doulas play in advocating for their clients when the white medical gaze filters out their precarious positionalities, I have added nuance to the existing literature on the importance of doula birth support for birthing women of color in the United States. However, it is still unknown to what extent CBDs can improve the birth experiences and outcomes for precarious women of color like the Central American migrants I began this article with, nor whether CBD work can affect long-term systemic change in US birth care. Future research could expand upon this study by investigating the perceptions and experiences that different precarious groups of women have with community-based doulas in the United States, and how CBDs continue to negotiate their role in birth care for women of color as they work within, alongside, or against state maternal health systems.

## Note

1. California’s branch of Medicaid, the joint federal and state health program that offers affordable medical care for people with limited income and resources who meet specific qualifications (DPSS 2024).
